# Comparison of the effect of high versus low mean arterial pressure levels on clinical outcomes and complications in elderly patients during non-cardiothoracic surgery under general anesthesia: study protocol for a randomized controlled trial

**DOI:** 10.1186/s13063-017-2233-8

**Published:** 2017-11-21

**Authors:** Anmin Hu, Yan Qiu, Peng Zhang, Bailong Hu, Yali Yang, Shutao Li, Rui Zhao, Zhongjun Zhang, Yaoxian Zhang, Zihao Zheng, Chen Qiu, Furong Li, Xiaolei Gong

**Affiliations:** 1 Department of Anesthesiology, Shenzhen People’s Hospital, Shenzhen, 518001 China; 2 Shenzhen Anesthesiology Engineering Center, Shenzhen, 518001 China; 3 The Second Clinical Medical College, Jinan University, Shenzhen, 518001 China; 4 Department of Anesthesiology and Translational Neuroscience Center, West China Hospital, Sichuan University, Chengdu, 610000 China; 5 Department of Anesthesiology, Sichuan Academy of Medical Sciences & Sichuan Provincial People’s Hospital, Chengdu, 610072 China; 6 Department of Anesthesiology, Affiliated Hospital of Guizhou Medical University, Guiyang, 550004 China; 7 Department of Anesthesiology, Henan Provincial People’s Hospital of Zhengzhou University, Zhengzhou, 450000 China; 8 Department of Anesthesiology, Taihe Hospital of Hubei University of Medicine, Shiyan, Hubei 442000 People’s Republic of China; 9 Department of Anesthesiology, The Third Affiliated Hospital of Kunming Medical University, Kunming, 650106 China; 10 Clinical Medical Research Center, The Second Clinical Medicine College, Jinan University, Shenzhen, 518001 China

**Keywords:** Mean arterial pressure, General anesthesia, Postoperative complication, Elderly patients

## Abstract

**Background:**

Intraoperative blood pressure (BP) is a concern in daily clinic anesthesia and contributes to the differences in clinical outcome. We conducted a randomized controlled trial (RCT) to compare the effect of high vs. low mean arterial pressure (MAP) levels on clinical outcomes and complications in elderly patients under general anesthesia (GA).

**Methods:**

In this multicenter, randomized, parallel-controlled, open-label, assessor-blinded clinical trial, 322 patients aged more than 65 years will be randomized for a low-level MAP (60–70 mmHg) or high-level MAP (90–100 mmHg) during non-cardiothoracic surgery under GA. The primary outcome will be the incidence of postoperative delirium. The secondary outcomes will include the delirium duration days, intraoperative urine volume, intraoperative blood loss, specific postoperative complications, and all-cause 28-day mortality.

**Discussion:**

Results of this trial will help clarify whether BP management is beneficial for elderly patients under GA and will make clear whether the effect of high-level MAP can reduce the postoperative complication compared to low-level MAP.

**Trial registration:**

ClinicalTrials.gov, NCT02857153. Registered on 15 July 2016.

**Electronic supplementary material:**

The online version of this article (doi:10.1186/s13063-017-2233-8) contains supplementary material, which is available to authorized users.

## Background

Population ageing worldwide is rapidly accelerating from 461 million people aged over 65 years in 2004 to an estimated 2 billion people by 2050 [1, 2]. Many studies in elderly persons show an association between the presence of low blood pressure (BP) and adverse outcomes, including increased cognitive impairment [3, 4], risk of dementia [5], and mortality [6, 7]. Periprocedural BP could have been influenced by anesthetic management and contributed to the differences in clinical outcome [8–12].

Hypotension has the potential to cause an ischemia-reperfusion injury which may manifest as dysfunction of any vital organ. Among the most sensitive organs to be affected in this way are the brain, kidney, and heart. Low intraoperative mean arterial pressure (MAP) is associated with increased postoperative delirium [13], risk of stroke [14], acute kidney injury [15], myocardial infarction [15, 16], and 30-day mortality [17–20]. In 101 femoral neck fracture patients aged more than 65 years under spinal anesthesia, patients who developed postoperative delirium (PD) had perioperative falls in BP after surgery [13]. A MAP < 60 mmHg and a relative decrease in MAP of 40% or more from the pre-induction BP is associated with postoperative myocardial injury if the decrease persists for more than 30 cumulative minutes [16]. MAP < 55 mmHg was associated with the development of postoperative acute kidney and myocardial injuries [15]. Terri et al. reported that a significantly increased risk of 30-day mortality with a MAP ≤ 49 mmHg or MAP decreased to more than 50% from baseline for ≥ 5 min [18].

An important aspect of intervention to decrease postoperative complications is intraoperative BP management under general anesthesia (GA). Hypotension is recognized as an important factor in postoperative complications, but what BPs are unsafe is unclear in elderly patients. We designed this study to conduct a randomized controlled trial (RCT) to compare the effect of high vs. low MAP level on clinical outcomes and complications in elderly patients under GA.

## Methods

### Trial design

This will be a multicenter, open-label, prospective RCT. Elderly patients will be included from seven centers, including Shenzhen People’s Hospital affiliated to Jinan University, West China Hospital affiliated to Sichuan University, The Affiliated Hospital of Guizhou Medical University, Taihe Hospital affiliated to Hubei University of Medicine, The Third Affiliated Hospital of Kunming Medical University, Sichuan Provincial People’s Hospital, and Henan Provincial People’s Hospital. All participants provided their written informed consent to participate in a RCT that examined the effects of low-level MAP (60–70 mmHg) vs. high-level MAP (90–100 mmHg) in elderly patients (aged 65 years or more) during non-cardiothoracic surgery under GA (trial registered at ClinicalTrials.gov under registration number: NCT02857153). MAP was calculated from the standard equation MAP = (2/3) diastolic blood pressure (DBP) + (1/3) systolic blood pressure (SBP) (in mmHg). We hypothesize high-level BP of the intervention for reducing the incidence of postoperative complications.

**Fig. 1:**
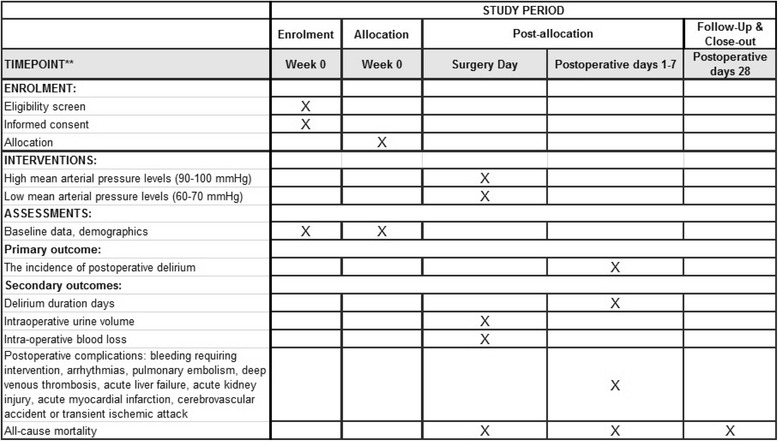
The schedule of enrollment, interventions, and assessments

This study protocol was written in accordance with the Standardized Protocol Interventions: Recommendations for Interventional Trials (SPIRIT) 2013 Statement (see Additional file 1 for the completed SPIRIT checklist) [21]. The time schedule of enrollment, assessment, interventions, and follow-up according to SPIRIT guidelines can be found in Fig. 1.

### Patient population and eligibility criteria

Patient eligibility is assessed at the preoperative anesthesia consultation. All eligible patients must sign a specifically designed informed consent form.

Inclusion criteria are: men and women; American Society of Anesthesiologists (ASA) I–II; aged 65 years or more; scheduled to undergo non-cardiothoracic surgery with GA; and 2 h or more of scheduled surgery time.

Participants will be excluded if they meet any of the following conditions: (1) the patient suffered from cardiovascular disease and metabolic diseases, such as hypertension, cardiac diseases, diabetes; (2) the patient has severe liver, kidney, or blood disease; (3) the patient is accompanied severe cognitive impairment (Mini-Mental State Examination [MMSE] score < 15) [22]; (4) preoperative history of schizophrenia, epilepsy, parkinsonism, use of cholinesterase inhibitor, or levodopa treatment; use of haloperidol or other neuroleptics during or after anesthesia; (5) neurosurgery; (6) individuals unlikely to survive for > 24 h; (7) previous participation in this study.

**Fig. 2:**
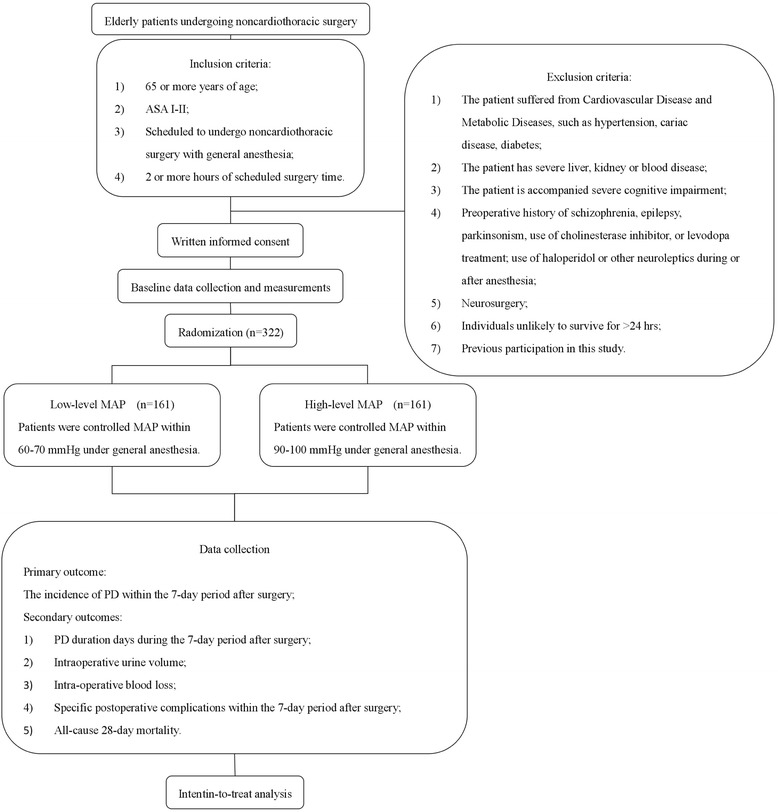
Trial flow chart

Trial schematic diagram is shown in Fig. 2.

### Randomization and blinding

Once consent is provided, participants will be assigned by the research assistant to either the low-level MAP group or high-level MAP group according to a random allocation sequence. An online random list generation will be utilized to perform blocked randomization in a 1 : 1 ratio. Trials cannot be blinded to anesthesiologist because anesthesiologist must know the MAP target to which each participant has been assigned in order to make the proper adjustment in the therapy to achieve 60–70 mmHg or 90–100 mmHg MAP. Outcome assessment and statistical analyses will be performed by independent researchers. Outcome assessor and the surgical team will be blinded to the study allocation status of participants.

### Intervention

On the day of surgery, patients come to the operating room and are provided with standard monitoring (electrocardiogram, oxygen saturation, non-invasive blood pressure, and invasive pressure monitoring). GA is given using midazolam (0.04 mg.kg^–1^) and propofol (as deemed appropriate by the anesthesiologist), opioids (fentanyl 3–6 μg.kg^–1^ and remifentanil 0.1–0.5 μg.kg^–1^.min^–1^), muscle relaxants (cisatracurium/rocuronium), and maintained with sevoflurane with inhaled concentrations of 1.5% sevoflurane in oxygen. Supplemental dosing of 1 μg.kg^–1^ of fentanyl is used every hour from induction up to approximately 1 h prior to the end of surgery. A tramadol bolus of 2 mg.kg^–1^ is given 15–30 min before the end of surgery. Propofol infusion is stopped 5–10 min before the end of surgery, whereas at the end of skin closure, remifentanil was discontinued.

According to grouping, MAP is regulated to the goal level (60–70 mmHg or 95–100 mmHg) during GA. If necessary, intravenous anti-hypertensives (urapidil [0.2–0.5 mg.kg^–1^] or phenylephrine [4–6 μg.kg^–1^] when MAP exceeds 10 mmHg of the target value), rather than more anesthesia, may be used in situations wherein sympathetic stimulation is high; yet a sufficient amount of anesthesia is administered and bispectral index (BIS) shows an adequate depth of hypnosis. Sedation is provided by a propofol infusion targeted to a BIS number of approximately 50 during GA. Atropine (5–10 μg.kg^–1^) and esmolol (1–3 mg.kg^–1^) would be used at the time of heart rate < 50 beats.min^–1^ and > 110 beats.min^–1^, respectively.

Lactated Ringer’s solution is given to bring the maintenance fluids to 10 mL.kg^–1^.h^–1^ [23]. Blood loss could be corrected for in a 1 : 1 ratio using gelofusine. Hospital transfusion guidelines are used to determine whether blood products are necessary (hemoglobin [HB] level < 10 g.dl^–1^ in patients with cardiac co-morbidities and < 7 g.dl^–1^ in those without cardiac disease). For later starting cases, an additional bolus of Ringer’s solution of 1.5 mL/kg/fasted hour from 08:00 _i_s given to bring the total to 2 mL/kg/fasted hour. If urine output decreases to < 0.5 mL.kg^–1^.h^–1^ for 1 h, fursemide 0.3 mg.kg^–1^ is given.

Mechanical ventilation patterns are adjusted to obtain an end-tidal carbon dioxide value of 35–45 mmHg at 5–10 min after induction of anesthesia.

For patients with endotracheal tubes, intravenous sedatives including propofol or midazolam were administrated continuously and titrated by bedside nurses to a target sedation level (Richmond Agitation Sedation Scale [RASS] range, – 2 to + 1) [24]. Daily awakening is used for those who were not extubated in the morning.

All patients receive patient controlled intravenous analgesia (PCIA) during postoperative days 1–3. Patients would be given PCIA with fentanyl 5 μg.mL^–1^, tramadol 5 mg.mL^–1^, and tropisetron 50 μg.mL^–1^. The PCIA is programmed to deliver a bolus dose of 2 mL, with background infusion of 2 mL/h and a lockout of 5 min, with a 1-h limit of 10 mL.

### Data collection

We will collect outcomes relating to four levels of specification of outcome measures as proposed by Zarin et al. [25]: domain (for example, postoperative delirium); specific metric (for example, RASS); specific metric used to characterize each participant’s results (for example, the incidence within the seven-day period after surgery); and method of aggregation (for example, mean change and 95% confidence interval).

A research assistant will collect date elements from patient medical records, including:

Demographic data:

- > data of birth;
- > gender;
- > preoperative diagnosis;
- > previous treatments;
- > co-morbid conditions;
- > preoperative vital signs: pulse (P), BP, respiratory rate (R);
- > baseline laboratory tests: aspartate transaminase (AST), alanine aminotransferase (ALT), blood urea nitrogen (BUN), serum creatinine (CREA), C-reactive protein (CRP), creatine kinase (CK), creatine kinase isoenzymes (CK-MB), brain natriuretic peptide (BNP), HB, white blood cell (WBC), platelet (PLT), oxygenation index (PaO_2_/FiO_2_), arterial carbon dioxide pressure (PaCO_2_), total bilirubin and fractions, prothrombin time (PT), activated partial thromboplastin time (APTT), international normalized ratio (INR);
- > preoperative electrocardiogram;
- > the ASA classification for preoperative health assessment of surgical patients [26].

Operative information:

- > name of surgical operation;
- > participation of fellow, resident, or medical student/clinical clerk in the surgical team;
- > intraoperative blood loss;
- > transfusion of blood products (packed red blood cells, platelets, fresh frozen plasma, albumin) and number of units;
- > volume of intravenous crystalloid infusion;
- > intraoperative urine volume;
- > intraoperative drug dosage, including in urapidil, phenylephrine and fursemide;
- > duration of surgery (defined as the number of hours between skin incision and closure of skin).

### Laboratory analyses

Preoperative values of laboratory tests (AST, ALT, BUN, CREA, CRP, CK, CK-MB, BNP, HB, WBC, PLT, PaO_2_/FiO_2_ and PaCO_2_) performed within the last week before surgery will be used as baseline values (before surgery). Additional blood samples will be collected on postoperative day 1 for serial laboratory evaluation.

### Postoperative complications

An independent board-certified physician will be the outcome assessor. He will be instructed about diagnostic criteria for specific postoperative complications in this trial. The outcome assessor will evaluate all participants until 28 days after surgery.

Diagnostic criteria for specific postoperative complications:

- > postoperative cognitive impairment defined by the confusion assessment method for the intensive care unit (CAM-ICU) [27]. First, level of sedation (level of arousal) was assessed using the RASS. If the patient was deeply sedated or was unarousable (– 4 or – 5 on the RASS), then assessment was stopped and repeated later, and the patient was noted as comatose. If RASS was > – 4 (– 3 through + 4), then assessment was continued to the next step. Second, delirium was diagnosed using CAM-ICU. It detects four features of delirium: acute onset of mental status changes or a fluctuating course; inattention; disorganized thinking; and altered level of consciousness. To have delirium diagnosed, a patient must display the first two aforementioned features, with either the third or fourth aforementioned feature;
- > hemorrhage: if requiring reoperation, radiological or endoscopic intervention; information on postoperative transfusion of blood products (type and number of units) will also be collected;
- > arrhythmias: any change in cardiac sinus rhythm prompting specific medical intervention or patient transfer to a monitored bed;
- > pulmonary embolism: confirmed by computed tomography (CT);
- > deep venous thrombosis: confirmed by Doppler ultrasonography;
- > acute liver failure: confirmed by physical exam, laboratory findings, patient history, and past medical history to establish mental status changes, coagulopathy, rapidity of onset, and absence of known prior liver disease, respectively [28];
- > acute kidney injury: defined by the Kidney Disease: Improving Global Outcomes (KDIGO) creatinine-based criteria [29];
- > all cases of acute myocardial infarction, cerebrovascular accident, or transient ischemic attack, as diagnosed by appropriate medical specialist;
- > other: any unlisted postoperative complications requiring specific medical treatment, radiological intervention or reoperation.

### Study outcomes

Primary outcome measure:

The incidence of PD within the seven-day period after surgery. Patients will be assessed for delirium with the CAM-ICU twice daily (at 08:00–10:00 and 18:00–20:00) during the first seven days after surgery.

Secondary outcomes:

1. Delirium duration days during the seven-day period after surgery.
2. Intraoperative urine volume.
3. Intraoperative blood loss: estimated using loss of red cell mass, which was derived from differences in pre- and postoperative hematocrits and transfused red cell mass with the following equation [30]: loss of red cell mass (mL)  =  estimated blood volume of patient (mL)  ×  (preoperative hematocrit (%) − immediate postoperative hematocrit (%))/100 + (transfused packed RBCs [unit]  ×  213  ×  0.7) (estimated blood volume of patient [mL]  =  75 mL/kg for men or 65 mL/kg for women  ×  body weight [kg]; 213 mL for average volume of packed RBC; 0.7 value for hematocrit of packed RBCs).
4. Specific postoperative complications within the seven-day period after surgery, including in bleeding requiring intervention, arrhythmias, pulmonary embolism, deep venous thrombosis, acute liver failure, acute kidney injury, acute myocardial infarction, cerebrovascular accident, or transient ischemic attack.
5. All-cause 28-day mortality.

### Adverse events

Serious adverse events and other untoward events that require hospitalization, are life-threatening, or result in death are collected following the SPIRIT recommendations [21]. All adverse events will be recorded and closely monitored until resolution or stabilization or until it has been shown that the study treatment is not the cause of the event. The Chief Investigator will be informed immediately of any serious adverse events and will determine (in cooperation with the treating medical practitioners) the seriousness and causality of these events. All treatment-related serious adverse events will be recoded and reported to the research Ethics Committee as part of the report. Unexpected serious adverse events will be reported to the research Ethics Committee within the relevant time frames. The Chief Investigator will be responsible for all adverse event reporting. All site staff will be appropriately trained in the procedures to follow and the forms to use during the study protocol before study initiation. If serious side-effects occur, the Chief Investigator can then unblended the participant and give the patient post-trial care.

### Withdrawal and dropout

Participation in the study will end at any stage if the patient refuses to continue, withdraws consent, or violates inclusion or exclusion criteria or the trial protocol. The trial will be stopped if the principle investigator believes that there are unacceptable risks of serious adverse events. No interim analysis will be conducted; however, the Data Safety and Monitoring Committee (DSMC) will perform regular reviews of all study outcome and adverse event data to ensure that there is no difference in rates of hospitalization or exacerbation in either group. The DSMC will determine final criteria for early study termination, which may be based on clear-cut evidence of worsened safety in one of the trial arms, and in the case of evidence beyond reasonable doubt of clear-cut benefit in the primary outcome measure, an effect size which would change clinical practice in the presence of the current literature and understanding of the disease area.

### Confidentiality

On recruitment, the research assistant will give a unique scrambled study number to each participant. Only the study number will be used to identify participants. Data collection sheets and any printout of electronic files will be kept in a locked filing cabinet in a secure office with limited access. The master list of participants and informed consent forms will be securely stored separately from de-identified participant records. All digital files will be password protected and stored in a firewall protected secure environment. The trial sponsor has access to the final trial dataset.

### Statistical analysis

Postoperative delirium is a frequent complication after major surgery in elderly patients and is related to an increase in mortality [31]. Probability of first postoperative day PD in elderly hip fracture patients is in the range of 20–60% [9]. PD occurred in 44% of elderly patients after a major operation [31]. Our sample size was calculated to detect a 15% difference in PD between the two groups with a two-tailed test, a significance level (alpha) of 5%, and a power of 80%. The sample size calculation was performed on STATA 14.0 software (StataCorp, College Station, TX, USA). We plan to include a total of 322 participants.

Analyses will be conducted at the Clinical Research Institute of Shenzhen People’s Hospital by blinded biostatisticians. Data analysis is on an intention-to-treat basis.

The level of significance was set at 5% and statistical analysis was performed using GraphPad Prism version 6.02 for Windows (GraphPad Software, San Diego, CA, USA). Continuous variable will be summarized using means and SDs (normally distributed data) or medians and ranges (non-normally distributed data). We will perform group comparison by using the Student’s t test or Mann–Whitney test for quantitative variables and Pearson’s chi-squared test for categorical variable. The odds ratio and 95% confidence interval will be computed with logistic regression.

### Trial oversight

A Trial Management Group including the Chief Investigator, Trial Coordinator, Trial Manger, Data Manager and Trial Statistician are in contact weekly.

A Trial Steering Committee (TSC) will meet at least three times each month over the Internet, and more frequently if required, to review the trial progress and to ensure that it is being conducted in accordance with the protocol, relevant regulations, and the principles of good clinical practice.

A DSMC will review trial progress and safety data. The DSMC is independent of the trial investigators and will comprise three independent members including two clinical specialists and a trial statistician.

### Protocol amendments

Protocol amendments will be agreed upon with the TSC, DSMC, Sponsor and Funding Body before submission for ethical approval. Following ethical approval, protocol modifications will be communicated with relevant parties such as the trial investigators, the trial registry, and, if required, trial participants.

### Dissemination policy

The results of the trial will be widely disseminated to patients, health professionals, commissioners, policy makers, and the general public. Our patient public involvement members will play a key role in this. The trial results will be disseminated to a wide clinical audience through publication in a high impact international scientific journal.

## Discussion

Intraoperative MAP influences clinical outcomes [8, 9, 32]. Intraoperative hypotension has been implicated in ischemia-reperfusion injury to the brain, heart, and kidneys and is associated with death [15, 17, 20]. This multicenter, open-label, randomized, parallel-controlled clinical trial is designed to test the hypothesis that, compared with low-level MAP, high-level MAP under GA reduces postoperative complications in elderly patients during non-cardiothoracic surgery. Even a neutral result will provide an important insight, as this would mean that more studies are needed to explore a safe and effective way of blood pressure management during GA in elderly patients. This is the main strength of the present study.

There are some limitations to our study protocol. First, a multicenter design is adopted so that patients enrolled in the study include a variety of surgical procedures under different clinical environments. This will increase the generalizability of our results. Because of the apparent difference between BP management, a double-blind design cannot be achieved. Therefore, we had to use an open-label design. The following measures will be undertaken to decrease the risk of potential bias. Second, there is evidence that intraoperative BP influence long-term consequences in elderly patients after GA [33]. However, our research is primarily concerned about the effects of low-level vs. high-level MAP on short-term postoperative complications. Third, patients who will be included as eligible participants should have no cardiovascular disease and metabolic diseases. Therefore, the results of this study will be restrainedly applied to all elderly patients during non-cardiothoracic surgery under GA.

Appropriate BP management would improve patient outcomes. Strengths of the protocol include that it is the first multicenter and centrally randomized study and that it observes both the primary (the seven-day incidence of PD) and secondary endpoints (duration days of PD, intraoperative urine volume, intraoperative blood loss, specific postoperative complications, and all-cause 28-day mortality). Results of the study will provide evidence for chasing felicitous BP management in elderly patients during non-cardiothoracic surgery under GA.

### Trial status

The trial is currently recruiting participants. To date (1 November 2016), 60 participants have been recruited. A report releasing study results will be submitted for publication in an appropriate journal, approximately 15 months after finishing data collection.

## Additional file

Additional file 1: SPIRIT checklist. Completed SPIRIT checklist. (PDF 97 kb)

## Abbreviations

ALT: Alanine aminotransferase
APTT: Activated partial thromboplastin time
ASA: American Society of Anesthesiologist
AST: Aspartate transaminase
BIS: Bispectral index
BNP: Brain natriuretic peptide
BP: Blood pressure
BUN: Blood urea nitrogen
CAM-ICU: Confusion assessment method for the intensive care unit
CK: Creatine kinase
CK-MB: Creatine kinase isoenzymes
CREA: Serum creatinine
CRP: C-reactive protein
CT: Computed tomography
DBP: Diastolic blood pressure
DSMC: Data Safety and Monitoring Committee
GA: General anesthesia
HB: Hemoglobin
INR: International normalized ratio
IRB: Institutional Review Board
KDIGO: Kidney Disease, Improving Global Outcomes
MAP: Mean arterial pressure
MMSE: Mini-Mental State Examination
P: Pulse
PaCO2: Arterial carbon dioxide pressure
PaO2/FiO2: Oxygenation index
PCIA: Controlled intravenous analgesia
PD: Postoperative delirium
PLT: Platelet
PT: Prothrombin time
R: Respiratory rate
RASS: Richmond Agitation Sedation Scale
RCT: Randomized controlled trial
SBP: Systolic blood pressure
SPIRIT: Standard Protocol Items, Recommendations for Interventional Trials
TSC: Trial Steering Committee
WBC: White blood cell
